# Imputation methods for mixed datasets in bioarchaeology

**DOI:** 10.1007/s12520-024-02078-2

**Published:** 2024-10-23

**Authors:** Jessica Ryan-Despraz, Amanda Wissler

**Affiliations:** 1 Department of Physical Anthropology, University of Bern, Bern, Switzerland; 2 Department of Anthropology, McMaster University, Hamilton, Canada

**Keywords:** Bioarchaeology, Missing data, Imputation, Mixed data

## Abstract

Missing data is a prevalent problem in bioarchaeological research and imputation could provide a promising solution. This work simulated missingness on a control dataset (481 samples × 41 variables) in order to explore imputation methods for mixed data (qualitative and quantitative data). The tested methods included Random Forest (RF), PCA/MCA, factorial analysis for mixed data (FAMD), hotdeck, predictive mean matching (PMM), random samples from observed values (RSOV), and a multi-method (MM) approach for the three missingness mechanisms (MCAR, MAR, and MNAR) at levels of 5%, 10%, 20%, 30%, and 40% missingness. This study also compared single imputation with an adapted multiple imputation method derived from the R package “mice”. The results showed that the adapted multiple imputation technique always outperformed single imputation for the same method. The best performing methods were most often RF and MM, and other commonly successful methods were PCA/MCA and PMM multiple imputation. Across all criteria, the amount of missingness was the most important parameter for imputation accuracy. While this study found that some imputation methods performed better than others for the control dataset, each imputation method has advantages and disadvantages. Imputation remains a promising solution for datasets containing missingness; however when making a decision it is essential to consider dataset structure and research goals.

**Supplementary Information:**

The online version contains supplementary material available at 10.1007/s12520-024-02078-2.

## Introduction

Missing data is a prominent problem across disciplines, not least of all bioarchaeology. Skeletal remains from archaeological contexts are rarely completely preserved, introducing questions of proper data treatment as well as having to contend with limited options for analysis.

There are three options when faced with the presence of missingness: analyze the dataset as is, delete samples or variables containing missingness, or impute the missing values. For the first option, attempting to analyze a dataset containing missingness is rife with problems, most notably the lack of options. Many methods, especially those performing multivariate analyses, are not possible with missing data. Even with regard to univariate analyses, such as basic hypothesis tests, there will always be the question of how the missing individual or variable could have influenced the outcome. Deletion is similarly unideal because the researcher loses information. For these reasons, the third option of data imputation is often a preferred solution. However, this adds an extra, time-consuming step to data analyses.

Bioarchaeologists widely recognize that missing data are a common feature of anthropological datasets and that this missingness can have important implications for the types of questions that can be asked, the analyses that can be performed, and the dependability of the conclusions. In their recent article, Wissler et al. (2022a) provided an overview of how missing data have been handled in bioarchaeology between 2011–2020. They found that out of 959 articles, only 267 engaged with missing data at all, suggesting that a large number of articles do not acknowledge the presence of missing data in their datasets. When missing data were engaged with, the authors found a predominance of less rigorous methods. The most common way bioarchaeologists managed missing values in their datasets was with some form of pre-analysis data treatments, such as substituting the right femur for the left or establishing a minimum threshold of completeness for inclusion in the study. Only 12% of the 267 articles (less than 5% of the total sample) managed missing data using advanced methods such as imputation. Wissler et al. (2022a) also found that the approach for handling missing data varied by subtopic within bioarchaeology. For example, biological distance and morphology studies were far more likely to use imputation compared to paleopathology or trauma studies.

One reason for this likely involves an overall lack of research into the applications, procedures, and limitations of imputing data in bioarchaeology (Wissler et al. 2022a). Some earlier studies focused on estimating individual vertebral heights or total vertebral column height when vertebrae are missing or damaged (Fully and Pineau 1960; Lundy 1985; Sciulli et al. 1990), and Auerbach et al. (2005) and Auerbach (2011) expanded upon the work of Fully (1956), designing regression formulas for estimating measurements for missing elements such as vertebral heights, femoral length, and tibial length. More recently, Kenyhercz & Passalacqua (2016) and Kenyhercz et al. (2019) tested many diverse forms of imputation methods on biological distance data on cranial metric and nonmetric data, assessing how different imputation methods impact the study results. Wissler et al. (2022b) likewise tested numerous imputation forms on bioarchaeological data alongside deletion methods to assess which method had the greatest success and to determine which deletion methods introduced the most bias.

Most recently, Pang and Liu (2023) evaluated the effectiveness of imputing craniometric and osteometric datasets by evaluating accuracy, robustness, and speed in 17 different methods. The studies assessing imputation methods in bioarchaeology (i.e. Kenyhercz and Passalacqua (2016), Kenyhercz et al. (2019), Wissler et al (2022a, b), and Pang and Liu (2023)) all drew somewhat different conclusions. Neither Pang and Liu (2023) nor Wissler et al. (2022a, b) were able to recommend a single imputation method that performed best across all the data. Kenyhercz & Passalaqua (2016) recommend k-nearest-neighbor imputation while Kenyhercz et al. (2019) found that iterative robust model-based imputation (IRMI) performed best across all their datasets.

Issues that complicate data imputation include mixed data (quantitative and qualitative variables in the same dataset), high-dimensionality (several variables, especially with respect to the number of samples), variable non-linearity, and generally complex relationships between the variables (Tang and Ishwaran 2017). In this regard, proper data imputation requires the researcher to have a thorough understanding of the data, notably the type of data, the type of missingness, the options for imputation methods, as well as the research question, before then choosing an appropriate imputation method.

### Type of data

Standard datasets, at least in anthropology, tend to be “rectangular” – a data matrix where the rows are samples (i.e. individuals or other, singular cases) and the columns are variables to be measured or scored (Little and Rubin 2002). Most imputation methods, and indeed most statistical analyses, are adapted to work with such matrices as well as particular types of data (variables) within them. Broadly speaking, this includes two main classifications: qualitative and quantitative. Qualitative data can be filtered into categories whereas quantitative data can be measured numerically (Lakshminarayan 2013; Ranganathan and Gogtay 2019). Further sub-categorizations of qualitative data include ordered, unordered (also commonly referred to as nominal), and binary. Ordered data contain observations that can be ranked (e.g. no bone fusion, fusion initiated, partial fusion, complete fusion), unordered data refer to unranked categorical classifications (e.g. blood type), and binary data are unordered data with only two categories (e.g. absent or present) (Lakshminarayan 2013; Ranganathan and Gogtay 2019). Quantitative data, also referred to as numerical data, has two types: discrete and continuous. Discrete data appear as whole numbers (e.g. number of teeth) whereas continuous data can be any value (e.g. femur length) (Lakshminarayan 2013; Ranganathan and Gogtay 2019). This study looks at mixed data, which includes both qualitative and quantitative data.

### Type of missingness

Missingness mechanism refers to whether or not missing data for a given variable are linked to the value. There are three classifications: Missing Completely At Random (MCAR), Missing At Random (MAR), and Not Missing At Random (MNAR)^1^ (Little and Rubin 2002; Rubin 1976; Schafer and Graham 2002). MCAR means that the missingness is *independent of both missing and present samples* – its individual value has nothing to do with the fact that it is missing. For example, a bioarchaeological dataset often has missing data due to a lack of bone preservation. In this case, measurements for the femur length and distal breadth might be missing – and indeed these two missing variables are linked (e.g. the distal femur is broken or missing thus limiting length measurements) – but this is independent of the sample (individual) and the measurement values themselves (e.g. the fact that femur length is immeasurable has nothing to do with the femur’s length). MAR data *depend on observed samples, not on missing samples*, and the values of the missing data are linked to the variable (Little and Rubin 2002; Schafer and Graham 2002). For example, when studying the dental profiles from a cemetery, there will be no third molar data obtained from young children. Therefore, the missingness in the data matrix for variables related to the third molar would be linked to certain observed samples (i.e. immature individuals), but not according to variation within the variable itself (i.e. individual parameters of the third molar have no bearing on missingness). Lastly, MNAR data is “nonignorable” and its *presence is linked to missing samples* (individuals) (Little and Rubin 2002; Schafer and Graham 2002) – in other words, the sources (i.e. samples) of the missing data are not observed. For example, if a researcher wants to gather paleopathological information about sex-based health differences in a past population by studying individuals interred in cemeteries, but the cemeteries did not include females, then the researcher has missing data for individuals they could not study. In some cases, MNAR data can be transformed into MAR data by removing problematic samples or variables, or even by changing the research question. Such adjustments could be necessary as many imputation methods are not intended for MNAR datasets.

In sum, data are MCAR if the probability of a value being missing is the same for all cases, data are MAR if the probability of being missing is related to the observed data, and data are MNAR if the probability of being missing is related to both observed and missing data.

### Imputation methods for mixed data

Imputation is the insertion of plausible values for missing values based on a predefined method. This often involves using observed data to create a predictive distribution and then drawing from the distribution to fill-in missing values (Little and Rubin 2002). The creation of these predictive distributions generally falls under two categories: explicit or implicit modeling. Explicit modeling bases the distribution on a model with explicit assumptions whereas implicit modeling is an algorithm (with an underlying model) with implicit assumptions (Little and Rubin 2002).

This work looks at seven imputation methods for mixed data, including: random forest (RF), PCA/MCA, factorial analysis for mixed data (FAMD), hotdeck, predictive mean matching (PMM), random samples from observed values (RSOV), and a multi-method approach (MM). For the intents and purposes of this study, each of these methods can be classified as either holistic or atomistic. The holistic methods, i.e. RF, FAMD, hotdeck, PMM, and RSOV, are technologically easier imputations to perform because the method takes into account the entire, mixed dataset at once. For the atomistic methods, i.e. PCA/MCA and MM, analyses require separate imputation calculations according to data type. From a programming point of view, the holistic methods are easier to perform as they require less coding and fewer parameters to consider. Depending on the method, additional advantages include the fact that imputed values are based on the entire dataset rather than just a subset. However, most datasets in bioarchaeology will have numerous dimensions as well complex relationships between variables, and some holistic imputation methods may become less accurate as complexity increases. In other words, a holistic method might be able to consider the entire dataset, but if that dataset is too complex, new problems surrounding precision may arise. For the atomistic methods, while they do not necessarily take into account the entire dataset and the relationships between all variables, they allow for more focused methods better adapted to a given data type.

#### Random forest (RF)

There are three common iterative (i.e. repetitive mathematical process) RF algorithms for imputing missing data, the first two being proximity (see Breiman 2003) – the data are pre-imputed to grow the forest and upon forest completion the pre-imputed values are updated using proximity – and an “on-the-fly” algorithm (see Ishwaran et al. 2008) where data imputation and forest-growing are simultaneous (Tang and Ishwaran 2017). This study focuses on the third common algorithm, missForest, due to its reported exceptional performance (Ramosaj and Pauly 2019; Stekhoven and Bühlmann 2012; Tang and Ishwaran 2017).

missForest works by first pre-imputing a missing value with either the variable mean or mode (Hong and Lynn 2020; Ramosaj and Pauly 2019; Stekhoven and Bühlmann 2012). Next, imputation is performed sequentially for each missing value such that each imputed value contributes to the imputation of the next. One iteration (a “tree” in the random forest, which this study set to the number of variables) is complete when all missing values have been imputed. This process then repeats until 1) the relative sum of squared differences (for quantitative data) or 2) the proportion of misclassifications (for categorical data) between iterations, increases (Hong and Lynn 2020; Stekhoven and Bühlmann 2012). The last iteration becomes the final result, and the researcher can set a limit for the maximum number of iterations. For the purposes of this work, RF is considered a “holistic” method because it is capable of handling mixed data without setting advanced, specific criteria according to data type.

RF imputations also provide an out-of-bag (OOB) error estimate, which uses the unselected training data to assess performance. This includes a normalized root mean square error (NRMSE) for numeric data and a proportion of correctly imputed values (PFC) for categorical data.

#### PCA/MCA

The PCA/MCA method imputes the numeric and categorical data separately and then re-combines them back into the same data set. These methods apply algorithms capable of performing PCA and MCA despite the presence of missing data and then use these components to reconstruct the data and impute the missing values (Audigier et al. 2016; Josse and Husson 2016). Due to the nature of the analyses, PCA assumes a linear relationship between variables and does not work well when variable relationships are too non-linear, whereas MCA does not require linearity as it focuses on finding patterns in the data. However, because MCA looks for patterns, it might not work as well if there are no associations between the categorical variables. Lastly, because PCA/MCA extracts only the most explanatory relationships in the data and therefore reduces dataset dimensions, it will be less successful if there are complex relationships between the variables. The R package missMDA also allows you to set the scale to either TRUE or FALSE based on whether or not the variables should have equal weights (this study set this parameter to TRUE).

#### Imputation by factorial analysis for mixed data (FAMD)

Like the PCA/MCA method, FAMD is also a principal component imputation method; however, this method has a scale step allowing it to impute mixed datasets (Audigier et al. 2016; Batbooti and Ransing 2023). This works by taking into account the type of data represented by each variable and then weighting it in order to balance the contributions of the categorical and the continuous variables (Audigier et al. 2016). By doing this, it ensures that each type of data is present in the structure of the principal components. This is essentially a type of low rank matrix completion algorithm (LRMC), meaning that the original data matrix is simplified (“low rank matrix”) based on underlying patterns in the data. Because of this underlying method, LRMC generally performs poorly on datasets with many samples but with few variables because the low-rank assumptions do not hold (low-rank assumptions assume the data is capable of being represented in fewer dimensions without losing information) (Zhao et al. 2022).

The FAMD calculation is more complex than for PCA/MCA because it has to consider both the categorical as well as the continuous variables – it needs to maintain both the structure of the categorical variables and the variance of the continuous variables – and it therefore may not be possible on datasets containing excessive missingness. What is considered “excessive” missingness will vary between datasets, however one rule of thumb would automatically exclude datasets containing > 30% missingness (Serneels and Verdonck 2008).

#### Hotdeck

Hotdeck imputation is a sampling-based method that works by replacing a missing value from a “donor” with similar observations (Kowarik and Templ 2016). This is usually applied in one of two ways. The first is random hotdeck in which several similar potential donors are identified and then one is randomly chosen to replace the missing value (used in this study). The second is nearest neighbor hotdeck in which a predetermined metric is used to establish a single sample as most similar to the case with the missing value to then apply the imputation (Andridge and Little 2010; Joenssen and Bankhofer 2012; Kowarik and Templ 2016). From a computational standpoint, the nearest neighbor approach is much slower.

When working with hotdeck, one risk is that unless the researcher defines advanced parameters, it could be difficult to know and trust what metrics the method used to identify donors. Along with this idea is also the problem that the same donor could be used to impute missing values for multiple samples (Joenssen and Bankhofer 2012). For some programs, it is possible to limit how many times the same donor is used, but this is also controlled for by using random hotdeck (Joenssen and Bankhofer 2012).

#### Random samples from observed values (RSOV)

RSOV is a straight-forward, sampling-based imputation method. This function imputes data by borrowing a value from the observed data without considering other conditions or variables (van Buuren and Groothuis-Oudshoorn 2011). Due to its simple nature, this will tend to be the least robust method and accuracy will decrease as diversity between the samples increases.

#### Predictive mean matching (PMM)

PMM is semi-parametric and related to the hotdeck method as imputed values are derived from observed cases (Ford 1983; Kleinke 2018; van Buuren and Groothuis-Oudshoorn 2011; Yang and Kim 2020). Broadly speaking, it is a two-step process that works by imputing a missing value from a donor with a similar predictive mean, making it a form of “nearest neighbor” imputation (Morris et al. 2014). The first step predicts regression values for both missing and observed values. The second step takes the predicted regression value of an observed data point that is most similar to that of a missing data point and uses the original observed value to impute the missing value (Hong and Lynn 2020; van Buuren 2018). The idea is to use a metric for matching missing values to observed values for imputation.

One drawback to PMM, and indeed other methods that require a donor, is the possibility that there is no donor with a predictive mean close enough to the sample containing missingness. This method may therefore not be ideal for smaller datasets (Bailey et al. 2020; Kleinke 2018; Morris et al. 2014; van Buuren 2018).

#### Multi-method approach (MM)

The MM approach applies different imputation methods according to data type, which for this study includes binary, ordered, unordered, and continuous. The four separate methods involved in this study include LASSO select + logistic regression for binary data, polytomous logistic regression for unordered data, a proportional odds model for ordered data, and LASSO select + linear regression for continuous data.

LASSO (least absolute shrinkage and selection operator) is a common method for building predictive linear models. Variables are weighted according to how likely they are to influence the outcome of a given prediction (Meier et al. 2008; Musoro et al. 2014; Rajaratnam et al. 2016; Tibshirani 1996). For example, when looking to predict the length of the radius, the length of the ulna would be more helpful than the width of the tibia. LASSO helps identify these important relationships by first giving a weight to each variable and then reducing their magnitudes until they reach zero, thus identifying them as less important and excluding them from the model (Andriopoulos and Kornaros 2023; Tibshirani 1996). The overall goal is to reach an equilibrium between having too many variables, thus risking a model that is too complex, and removing potentially important variables, which would make the model too simple.

It is important to underline the functional difference between dimension reduction as seen with LASSO versus with PCA and/or MCA. LASSO emphasizes variable selection from a large pool of predictors and simplifies analyses by removing data deemed extraneous while PCA/MCA takes datasets with high-dimensionality, finds important patterns within it, and then creates a representation of the same data in fewer dimensions (Audigier et al. 2016; Tibshirani 1996). While both methods aim to simplify a dataset by reducing dimensionality, LASSO does so by removing elements deemed irrelevant to create a predictive model, whereas PCA/MCA compacts the information contained within the dataset in order to facilitate analyses and visualizations. Both methods complement each other, yet each serves a distinct purpose.

Polytomous logistic regression (PLR) uses a multinomial model (outcome prediction with more than two categories) to relate categorical data to a set of predictor variables, which are then used to calculate the probability of a given outcome for the missing values (Engel 1989; Miron et al. 2022; van Buuren and Groothuis-Oudshoorn 2011; Venables and Ripley 2002). This method can handle multiple categories, however it requires sufficient data; according to van Buuren (2018), PMM could be preferable for smaller datasets, though it is still likely superior to discriminant analysis (Brand 1999). The proportional odds model (POM) is similar to PLR, though since it is used for ordered data, it also takes into account the distance between categories. Broadly speaking, POM calculates the cumulative odds of a value being less than or equal to or more than a particular level of a given ordinal variable (Hosmer et al. 2013; Liu et al. 2018). While a POM assumes that the odds of transitioning from one level to another are proportional, the R package “mice” (“polr” function) applies an ordered logit model, which does not assume that the odds of moving between ordered categories is proportional (Hosmer et al. 2013; van Buuren and Groothuis-Oudshoorn 2011). In this case, it estimates specific criteria for each ordered transition allowing for variations in the relationships between the predictors (McCullagh 1980; McCullagh and Nelder 1989; Venables and Ripley 2002). This could be useful for certain anthropological analyses because while some data may be ordered (e.g. age groups), the link to other factors (e.g. the presence of degenerative joint diseases) may vary throughout the categorical transitions.

### Single and multiple imputation

In general, the goal is to obtain a single, complete dataset that can be analyzed, which is why single imputation is quite popular. Single imputation (SI) assigns a single, imputed value for each missing value (Little and Rubin 2020). This is a straightforward approach, as well as computationally quicker than multiple imputation, however it is unable to account for uncertainties associated with the imputed values. In this sense, SI is more likely to underestimate standard error and therefore not fully represent a dataset’s variability (Kowarik and Templ 2016; Little and Rubin 2020). Multiple imputation (MI) controls for this issue by creating multiple, imputed datasets each with differing imputed values (Horton and Kleinman 2007; Little and Rubin 2020; Raghunathan et al. 2001; van Buuren et al. 2006; van Buuren and Groothuis-Oudshoorn 2011). Most commonly, this involves the iterative imputation of values while considering relationships between variables. It is then possible to perform statistical analyses or build models based on these multiple datasets. For these reasons, multiple imputation methods tend to perform better and thus be preferable; however, they also tend to be computationally demanding and require a broader understanding of the data structure and research objectives because, unlike with SI, the goal of MI is not to provide the researcher with a single, complete dataset that can then be analyzed. However, a single dataset was the goal of this work, which is why this study decided to apply an adapted MI method.

**Fig. 1:**
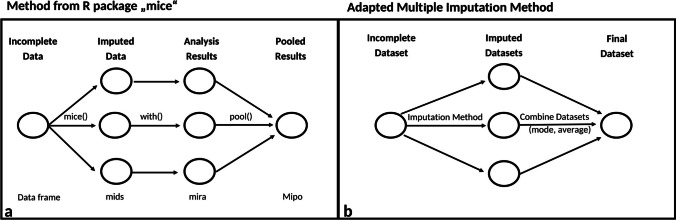
**a** The underlying method of multiple imputation in the R package “mice” (multiple imputation by chained equations), from van Buuren & Groothuis-Oudshoorn 2011, **b** The adapted multiple imputation method used in this study to achieve a single dataset

The “mice” R package (multiple imputation by chained equations) is a common and reliable tool for MI. MI in mice involves three primary steps: imputing multiple datasets, running a specific statistical analysis on each, and pooling the resulting test statistics (Fig. 1) (van Buuren and Groothuis-Oudshoorn 2011). With chained equations, the imputation model is specific to each variable, using other variables as predictors, and each newly imputed value is used in the predictor model imputing the next value and so on (hence the “chained” equation) (Horton and Kleinman 2007). MI methods using chained equations can therefore have a different “chain” for each individual imputation (see Horton and Kleinman 2007; Raghunathan et al. 2001; Van Buuren et al. 2006).

This study applied an adapted MI method based on mice with the goal of obtaining a final, complete dataset for the three imputation methods calculated in this R package: PMM, RSOV, and MM. This method applied the same framework as mice, but instead of pooling the results of a predetermined statistical test, it “pooled” the multiple datasets. In other words, it combined the multiple, imputed datasets into a single, master dataset by calculating either the mean (quantitative data) or the mode (qualitative data) (Fig. 1). The idea is that combining multiple, imputed datasets will minimize the effects of potentially outlying, poorly imputed values, thus making them more reliable than single imputations.

### Research objective

This study takes a complete (no missing data) mixed bioarchaeological dataset, simulates missingness, then performs various methods of data imputation. These imputed datasets are compared to the original dataset and the imputation methods evaluated for performance. This study also discusses aspects of dataset structure together with advantages and disadvantages of the given imputation methods with the goal of providing a starting point for bioarchaeologists looking to perform data imputations on mixed datasets.

## Materials and methods

All imputations and statistical analyses were performed in R version 4.3.1 with a seed set to 99. The original test dataset containing no missingness is available in the Supplementary Information (Table A) and all codes, the original dataset, and the datasets containing missingness are available on Github (https://github.com/JRyanDespraz/Bioarchaeology-Imputation). When importing a dataset into R, it is important to verify that the program recognizes the type of data represented by each variable, and for ordered categorical variables, it could be necessary to set the levels. For all tests applying multiple imputation using mice, the number of imputations (m) was set to the percent missingness; for example, a dataset with 10% missingness performed ten imputations (see Bodner 2008; White et al. 2011).

### The control dataset

The control group consisted of 481 individuals from the Hamann-Todd Documented Skeletal Collection in Cleveland, USA. There were a total of 41 variables, including for the categorical variables two binary, four ordered, and three unordered, and 32 continuous variables (Supplementary Information Table A). The binary data included sex (male or female) and ancestry (Black or White), the unordered data included lesion status (cribra orbitalia, porotic hyperostosis, and periosteal lesions of the tibia), the ordered data included age (organized into five groups, adults only) and lesion severity, and the continuous data included measurements of femoral length and vertebral neural canal transverse and anterior–posterior width. Note that the data have been altered slightly from the original to preserve the privacy of the deceased and the intellectual property rights of the Cleveland Museum of Natural History, who owns the Hamann-Todd data.

### Generating missingness

This study generated missingness at rates of 5%, 10%, 20%, 30%, and 40% for MCAR, MAR, and MNAR on the control dataset. These datasets were produced using the “missMethods” package in R, with the functions “delete_MCAR”, “delete_MAR”, and “delete_MNAR” (Rockel 2022). For MAR datasets, the pattern of missingness for each variable is dependent upon other variables within the dataset. The relationships were randomly assigned (e.g. missingness in Sex was dependent upon T1.AP measurements), and the percentage of missing data set to the desired amount. For the MNAR datasets, missingness was simulated in all columns and the percentage of missing data likewise set to the desired amount.

### Imputation methods

**Fig. 2:**
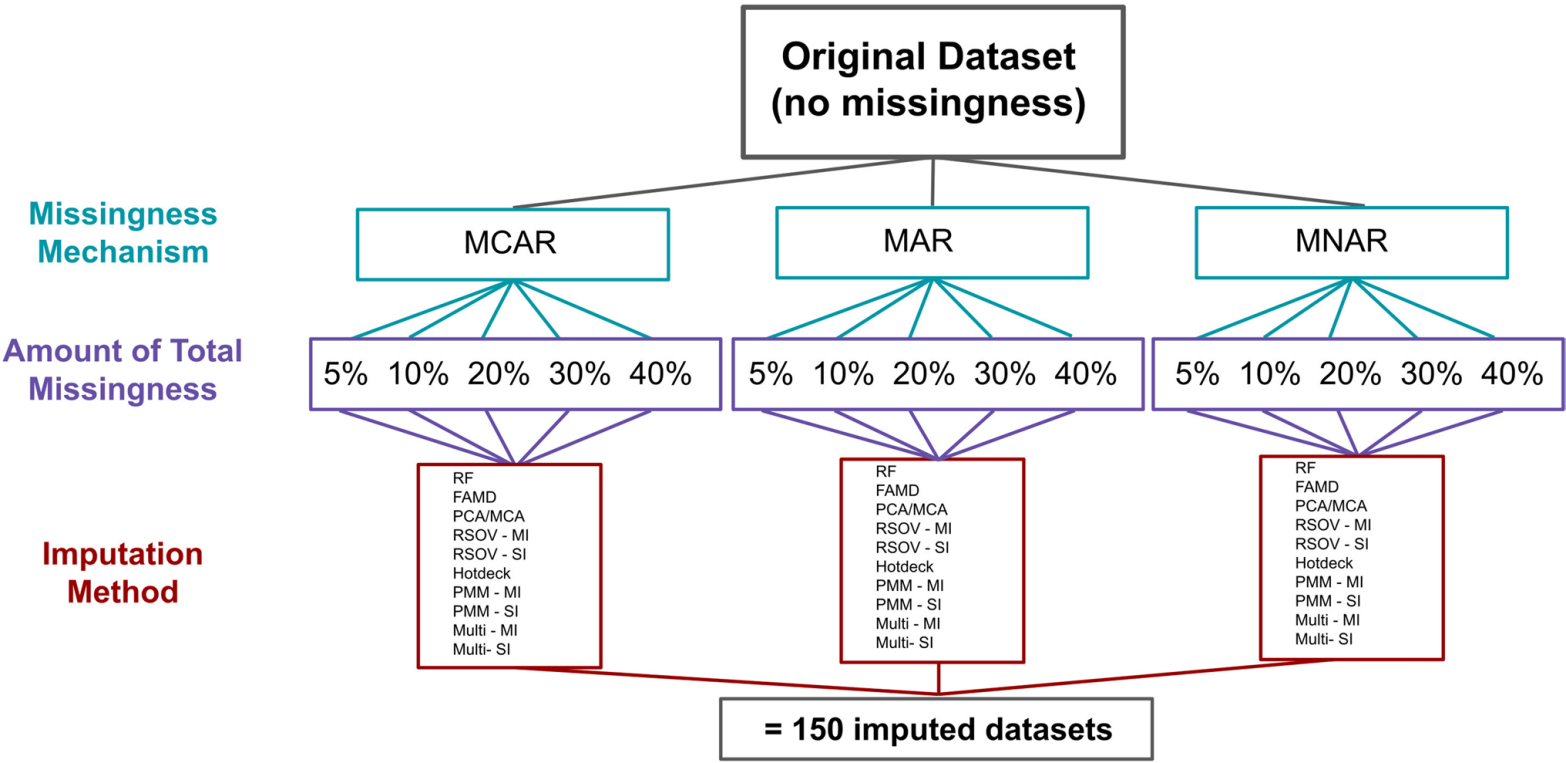
An overview of the steps taken to analyze missingness and imputation methods using a control dataset; MCAR = missing completely at random, MAR = missing at random, MNAR = missing not at random, RF = random forest, FAMD = factorial analysis for mixed data, RSOV = random samples from observed values, PMM = predictive mean matching, MM = multi-method approach, SI = single imputation, MI = multiple imputation

Each imputation method was performed on each dataset with the generated missingness. Three of the seven methods – RSOV, PMM, and MM – included both single and the adapted multiple imputation method. The three missingness mechanisms (MCAR, MAR, and MNAR), the five levels of missingness (5%, 10%, 20%, 30%, and 40%), and the seven imputation methods (three of which performed both SI and MI) creates a total of 150 imputed datasets (Fig. 2). The number of iterations was set to 50 for methods using mice and 1000 for PCA/MCA and FAMD. These latter two methods (applying dimension reduction) require the calculation of the number of components (ncp) before performing imputation. This parameter finds the optimal number of dimensions that should be maintained in the final analysis, which is based on the eigenvalues, and is necessary for finding the right balance to avoid overfitting or underfitting. It can be calculated in the same package (missMDA) using the functions estim_ncpPCA, estim_ncpMCA, or estim_ncpFAMD (Josse & Husson 2016). Table 1 presents an overview of each of the R packages and functions used for each imputation method.

**Table 1:** A summary of the R codes applied to perform each imputation method; additional information is available in the CRAN repositories; RF = random forest, FAMD = factorial analysis for mixed data, RSOV = random samples from observed values, PMM = predictive mean matching, MM = multi-method approach

Imputation Method	R package	R function	Additional Arguments	Source
Random Forest (RF)	missForest	missForest	maxiter, ntree	Stekhoven & Bühlmann 2012
PCA/MCA	missMDA	imputePCA, imputeMCA	estim_ncpPCA, estim_ncpMCA, maxiter, scale	Josse & Husson 2016
FAMD	missMDA	imputeFAMD	maxiter	Josse & Husson 2016
Hotdeck	VIM	hotdeck		Kowarik & Templ 2016
RSOV	mice	mice	method ("sample"), m, maxiter	van Buuren & Groothuis-Oudshoorn 2011
PMM	mice	mice	method ("pmm"), m, maxiter	van Buuren & Groothuis-Oudshoorn 2011
MM	mice	mice	method (“lasso.select.logreg”, “polyreg”, “polr”, and “lasso.select.norm”), m, maxiter	van Buuren & Groothuis-Oudshoorn 2011

### Assessing success

Each imputed dataset was compared to the original dataset. Calculations for the categorical data include misclassification error (ME) for the binary and unordered data and the mean absolute error (MAE) for ordered data (Zhao et al. 2022). Due to the fact that they had different ordered levels, the MAE for age and lesion severity data were calculated separately. Three calculations assess the continuous data: relative MAE, normalized root mean square error (NRMSE) of the column mean square, and the NRMSE of the total dataset standard deviation (Batbooti and Ransing 2023; Kim et al. 2005; Nienkemper-Swanepoel et al. 2023; Rockel 2022). The relative MAE expresses the MAE as a percentage of the average of the true values, which allows researchers to read the error with respect to the scale of the data. By squaring the differences of the original and imputed column means, the NRMSE (“NRMSE_col_mean_sq”) gives weight to larger deviations, thus emphasizing outliers. Since this study aims to assess the performance of imputation methods, this metric was deemed useful for quantifying larger errors. Lastly, the NRMSE of the total standard deviation (“NRMSE_tot_sd”) provides a comprehensive measure that considers both the mean and the variability of the entire dataset. These assessments were performed in R using the missMethods package and the function “evaluate_imputed_values()” (Rockel 2022).

In order to further assess success between the three missingness mechanisms, a final estimate calculated the average for each imputation method at every missingness level and data type (e.g. average[MCAR_5%, MCAR_10%, MCAR_20%, MCAR_30%, MCAR_40%] for RF binary data ME results). This provided a single value (Supplementary Information Table C) that then allowed for comparisons of each missingness mechanism.

Data output for all RF imputations also includes two out-of-bag (OOB) error estimates: a normalized root mean square error (NRMSE) for numeric data and a proportion of correctly imputed values (PFC) for categorical data.

## Results

A complete table of all calculated results for each of the 150 imputed datasets can be found in the Supplementary Information (Table B).

### Missingness mechanism

**Fig. 3:**
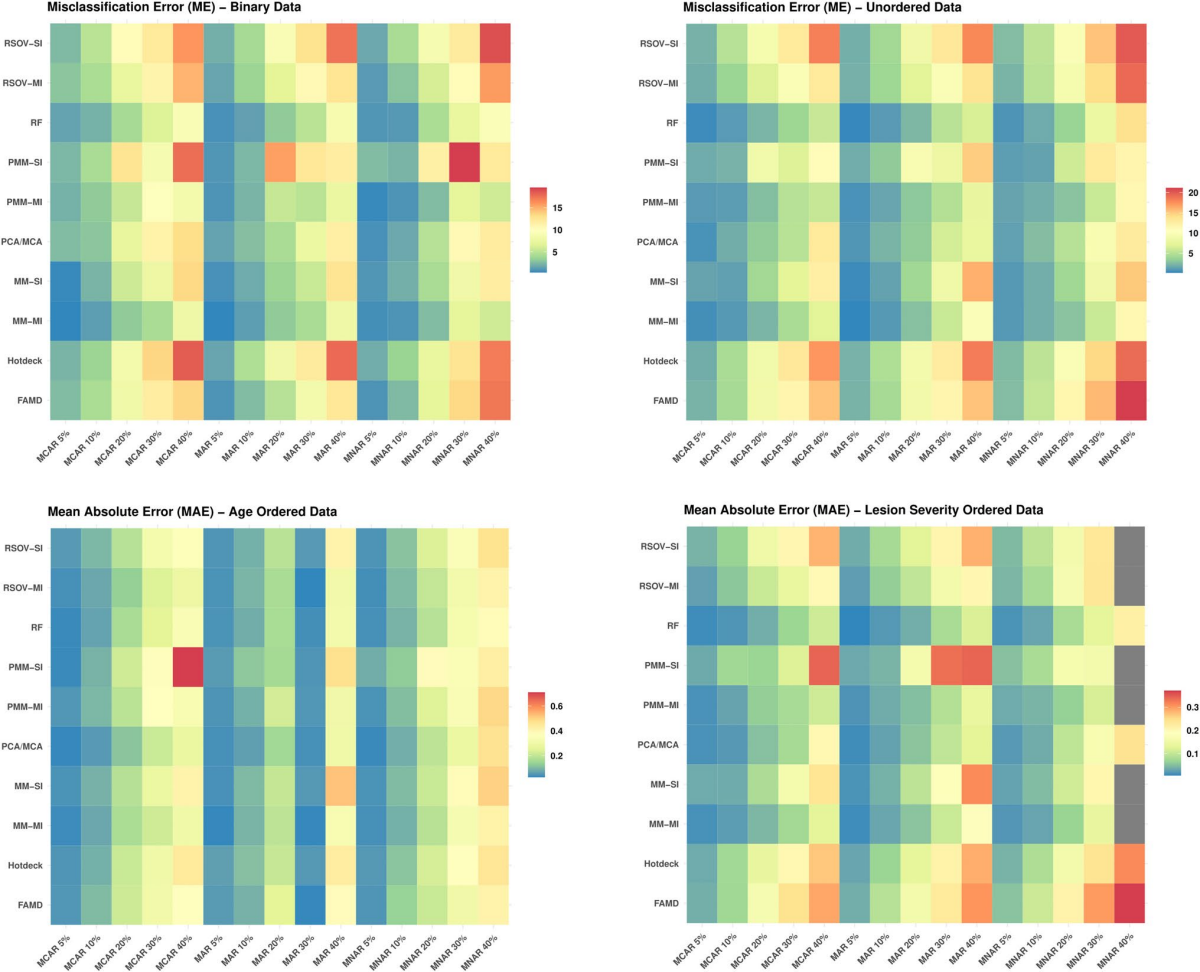
Heatmaps showing the analysis results of the categorical data (Supplementary Information Table B) for each imputation method according to data type, gray boxes indicate either an N/A result (MAE ordered data results) or an outlier that was removed for data visualization purposes; MCAR = missing completely at random, MAR = missing at random, MNAR = missing not at random, RF = random forest, FAMD = factorial analysis for mixed data, RSOV = random samples from observed values, PMM = predictive mean matching, MM = multi-method approach, SI = single imputation, MI = multiple imputation

**Fig. 4:**
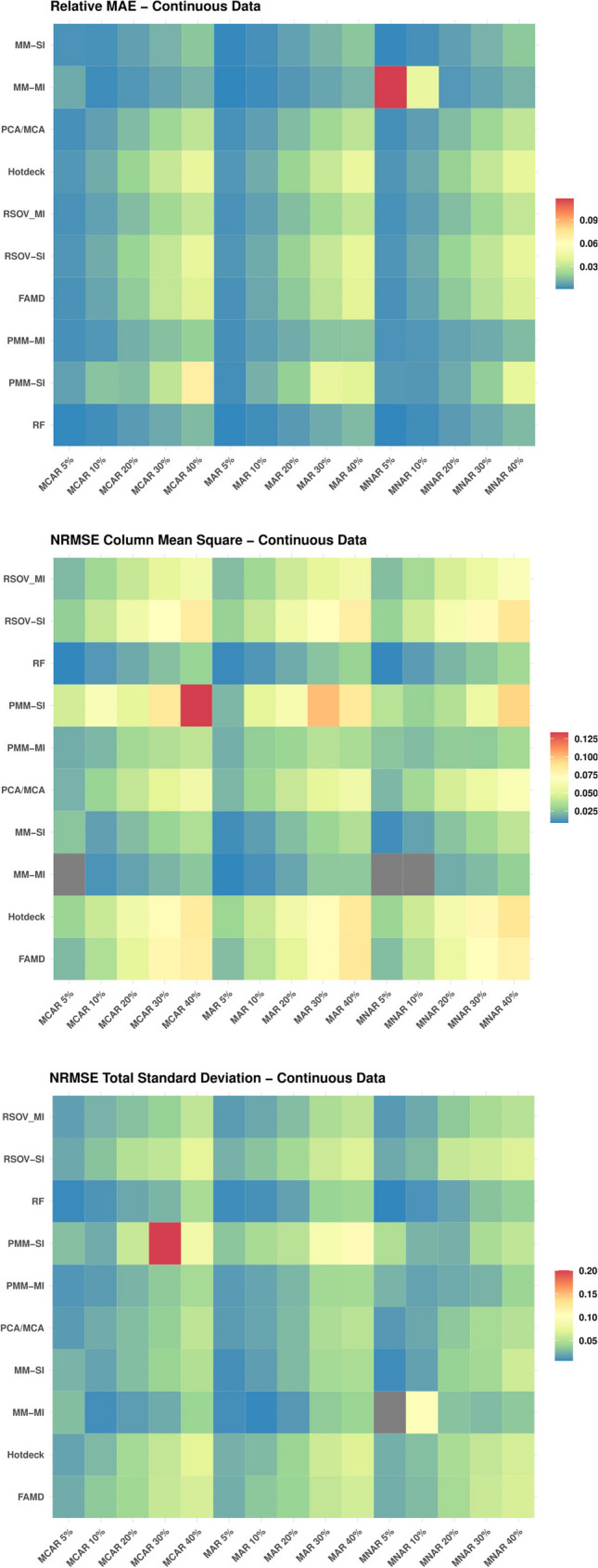
Heatmaps showing the analysis results of the continuous data (Supplementary Information Table B) for each imputation method according to data type, gray boxes indicate either an N/A result or an outlier that was removed for data visualization purposes (NRMSE); MCAR = missing completely at random, MAR = missing at random, MNAR = missing not at random, RF = random forest, FAMD = factorial analysis for mixed data, RSOV = random samples from observed values, PMM = predictive mean matching, MM = multi-method approach, SI = single imputation, MI = multiple imputation, NRMSE = Normalized Root Mean Square Error

Overall, there are few patterns in the data, with the exception of the ordered age data for which MAR was consistently the most accurate. In general, the categorical data imputations tended to work best on MCAR and MAR data; however, the continuous data results were much more variable, with many more instances of MNAR data providing the most accurate imputation accuracy (Table 2). The continuous data also provided many more instances where results were the same across all missingness mechanisms. This pattern is also visible in the heatmaps (Figs. 3 and 4).

**Table 2:** The missingness mechanism that provided the most accurate imputation result according to imputation method and data type, calculated based on an average of missingness levels (e.g. average[MCAR_5%, MCAR_10%, MCAR_20%, MCAR_30%, MCAR_40%] for RF binary data misclassification error results); MCAR= missing completely at random, MAR = missing at random, MNAR = missing not at random, RSOV = random samples from observed values, PMM = predictive mean matching, SI = single imputation, MI = multiple imputation, rel_MAE = relative mean absolute error, NRMSE_col_mean_sq = normalized root mean square error column mean square, NRMSE_tot_sd = normalized root mean square error total standard deviation

	Categorical Data				Continuous Data		
Imputation Method	Binary	Unordered	Ordered Age	Ordered Lesion	rel_MAE	NRMSE_col_mean_sq	NRMSE_tot_sd
Random Forest	MAR	MCAR	MAR	MCAR	MCAR/MAR/MNAR	MCAR/MAR	MNAR
FAMD	MAR	MCAR/MAR	MAR	MCAR	MNAR	MNAR	MNAR
Hotdeck	MNAR	MCAR	MAR	MCAR	MNAR	MNAR	MNAR
PCA/MCA	MAR	MAR	MAR	MAR	MNAR	MNAR	MAR
PMM—MI	MNAR	MCAR	MAR	MCAR	MNAR	MCAR/MAR	MAR
RSOV—MI	MAR	MCAR	MAR	MCAR	MCAR/MAR/MNAR	MCAR	MCAR/MAR
PMM—SI	MAR	MCAR	MAR	MNAR	MNAR	MCAR/MAR	MCAR/MAR/MNAR
RSOV—SI	MAR/MNAR	MAR	MAR	MNAR	MCAR/MAR/MNAR	MCAR	MCAR/MAR
Multi-Method—MI	MNAR	MCAR	MAR	MCAR	MAR	MAR	MAR
Multi-Method—SI	MAR	MCAR	MAR	MNAR	MCAR/MAR/MNAR	MAR	MAR

There were a few noteworthy outliers in the MM-MI imputation results for continuous MNAR data (Fig. 8 and Supplementary Information Table B). MM-MI (LASSO select + linear regression for continuous data) performed extremely poorly for MNAR datasets with 5% and 10% missingness. There was also an outlying value for the NRMSE_col_mean_sq for the MCAR dataset at 5% missingness.

### Amount of missingness

**Fig. 5:**
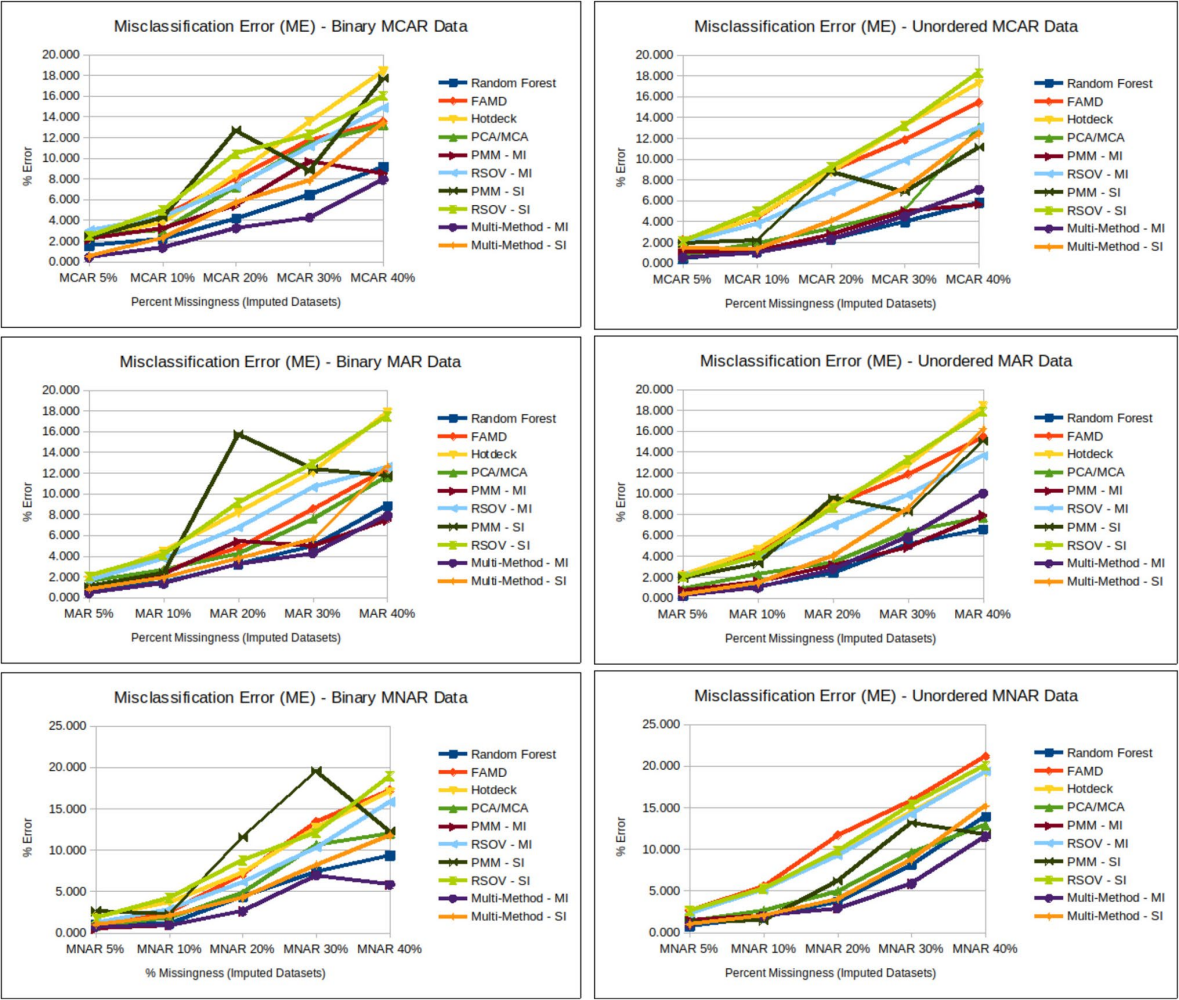
Line charts showing analysis results for ME (misclassification error) (Supplementary Information Table B) for each imputation method according to data type; MCAR = missing completely at random, MAR = missing at random, MNAR = missing not at random, RF = random forest, FAMD = factorial analysis for mixed data, RSOV = random samples from observed values, PMM = predictive mean matching, MM = multi-method approach, SI = single imputation, MI = multiple imputation

**Fig. 6:**
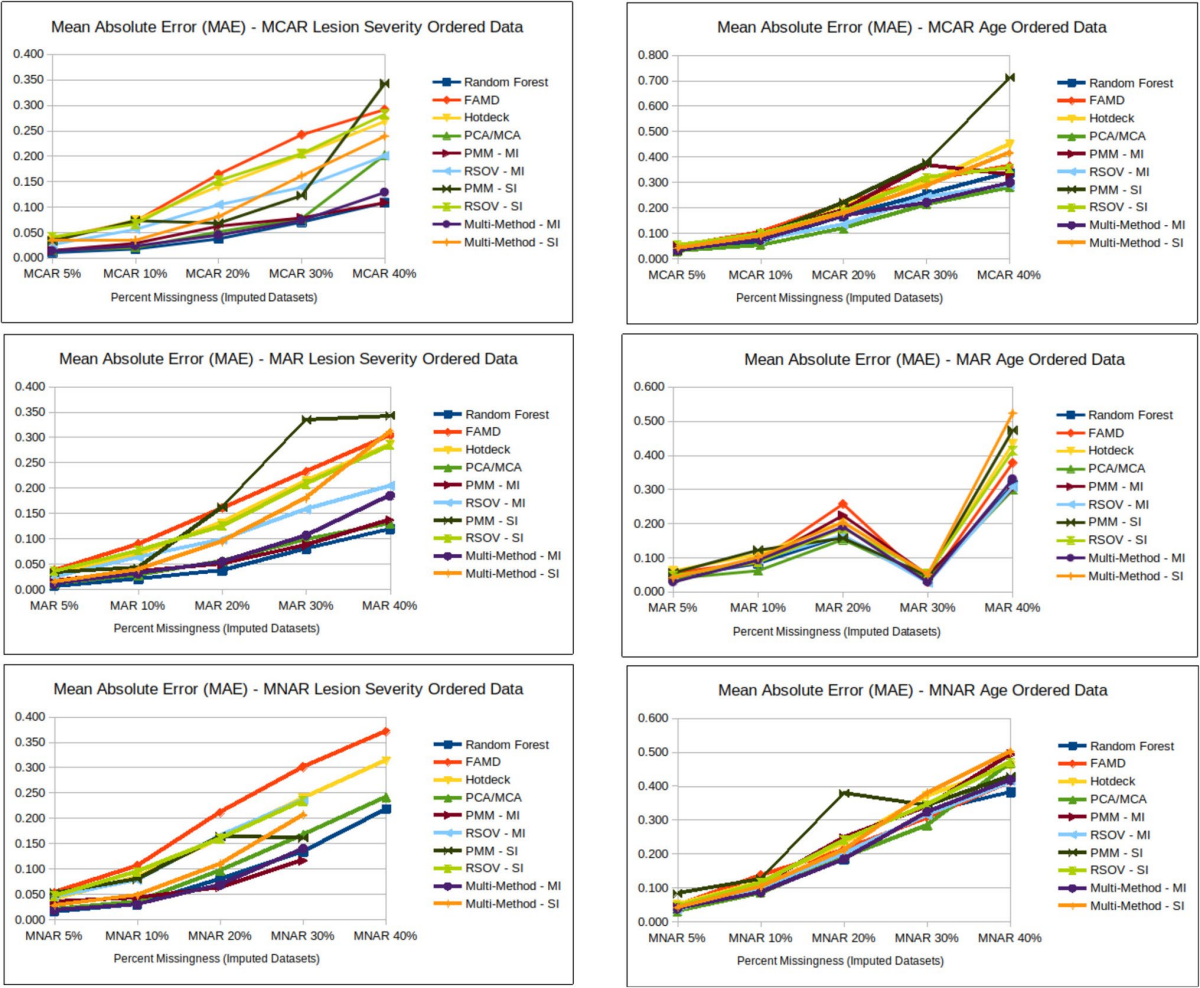
Line charts showing analysis results for MAE (mean absolute error) (Supplementary Information Table B) for each imputation method according to data type; MCAR = missing completely at random, MAR = missing at random, MNAR = missing not at random, RF = random forest, FAMD = factorial analysis for mixed data, RSOV = random samples from observed values, PMM = predictive mean matching, MM = multi-method approach, SI = single imputation, MI = multiple imputation

**Fig. 7:**
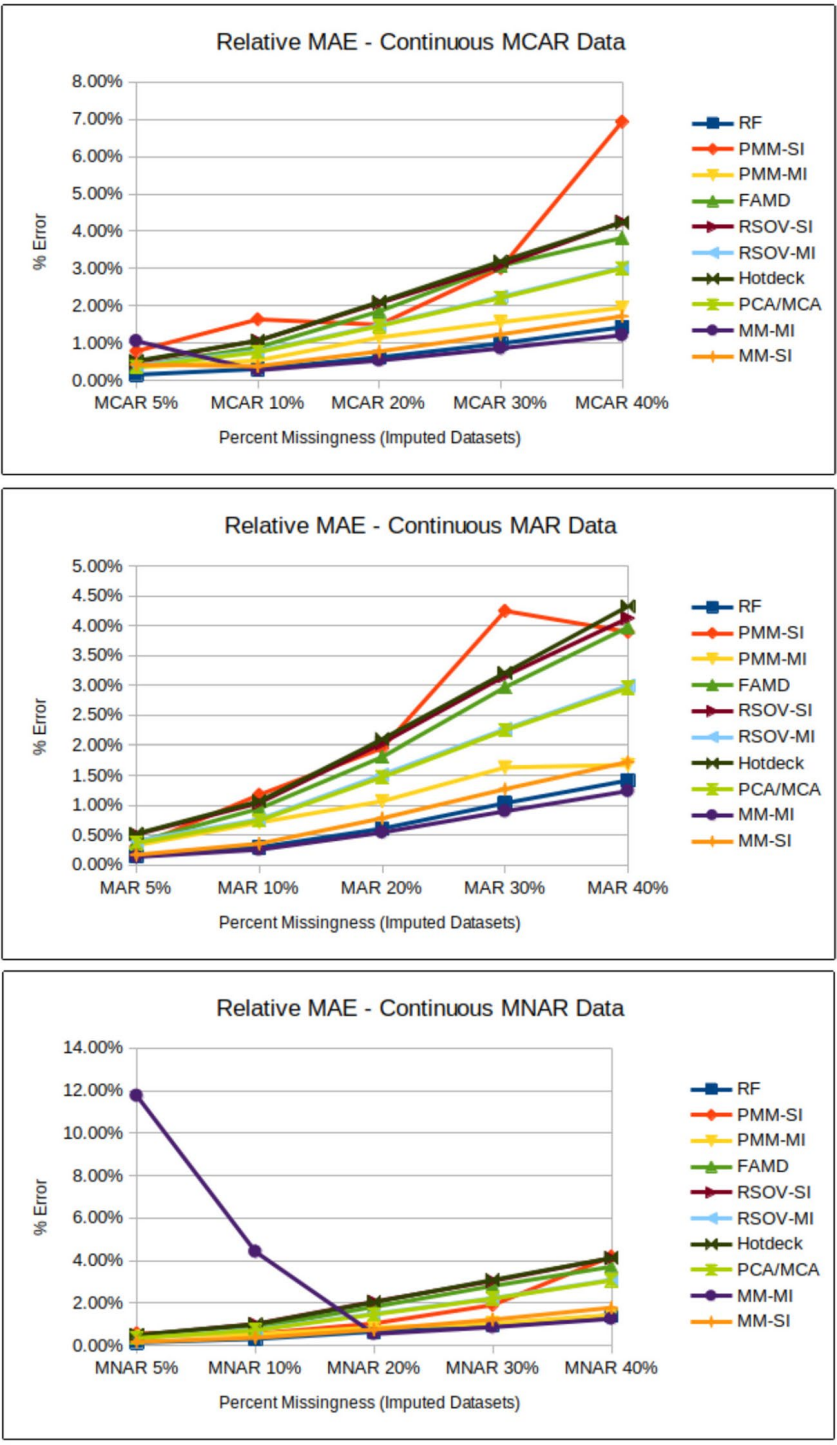
Line charts showing analysis results for relative MAE (mean absolute error) (Supplementary Information Table B) for each imputation method according to data type; MCAR = missing completely at random, MAR = missing at random, MNAR = missing not at random, RF = random forest, FAMD = factorial analysis for mixed data, RSOV = random samples from observed values, PMM = predictive mean matching, MM = multi-method approach, SI = single imputation, MI = multiple imputation

**Fig. 8:**
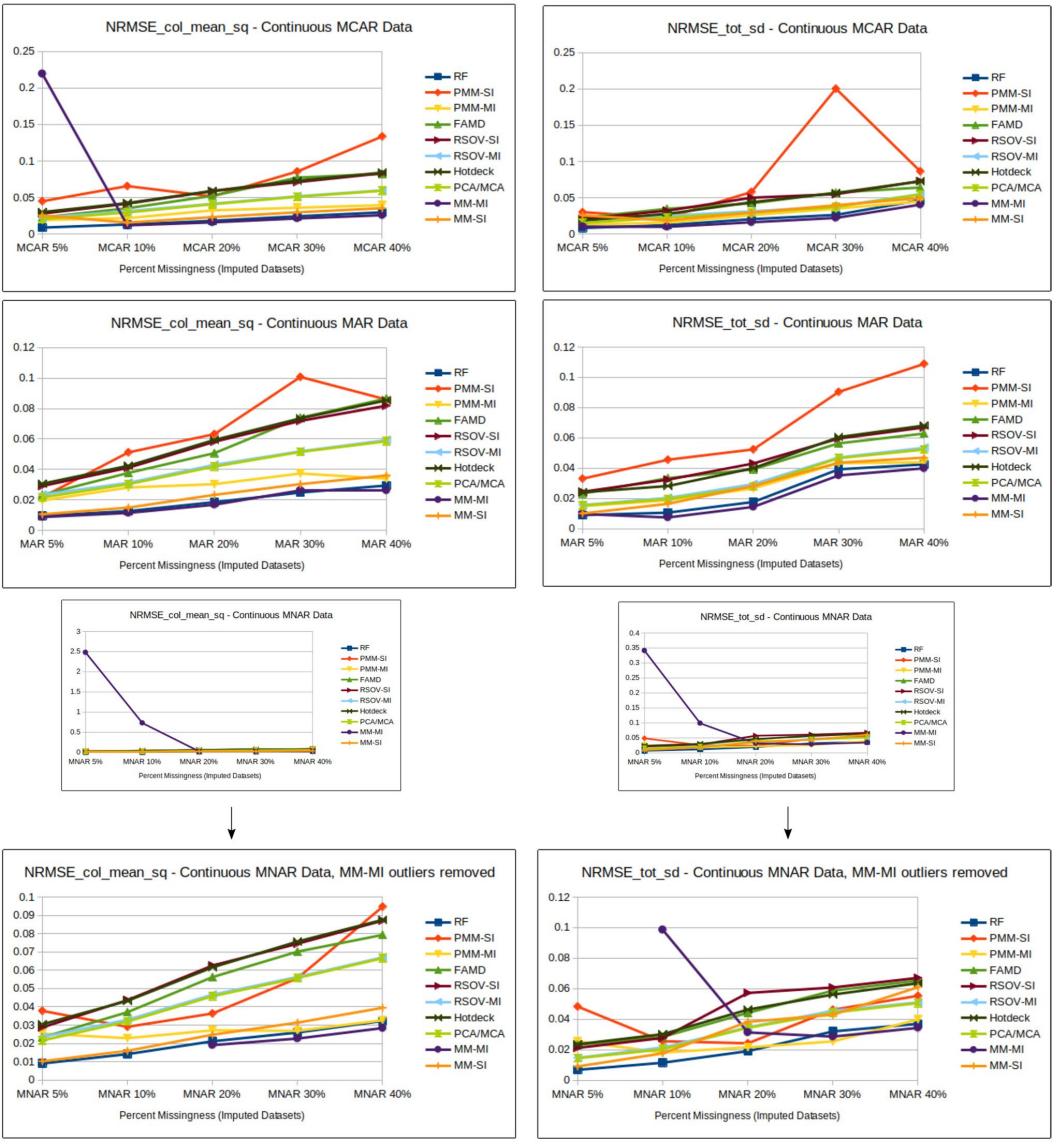
Line charts showing analysis results for NRMSE (normalized root mean square error) of the column mean squares and total standard deviation (Supplementary Information Table B) for each imputation method according to data type; MCAR = missing completely at random, MAR = missing at random, MNAR = missing not at random, RF = random forest, FAMD = factorial analysis for mixed data, RSOV = random samples from observed values, PMM = predictive mean matching, MM = multi-method approach, SI = single imputation, MI = multiple imputation

A consistent result across all three missingness mechanisms and all imputation methods is that performance decreases as missingness increases, though certain methods did perform better overall than others (Figs. 3 and 4). This case study shows that across all data types, there was little difference in imputation accuracy between 5 and 10%, but error increased steeply for percentages of missingness greater than 20% (Figs. 5, 6, 7 and 8).

One outlying phenomenon involved the MAE ordered age data calculation for MAR data at 30% missingness (Figs. 3 and 6). All methods found highly accurate imputations with an accuracy similar to findings at 5% missingness. Upon this initial finding, the original datasets were confirmed and all imputations were re-calculated with the same result.

### Imputation method

Comparing single (SI) versus the adapted multiple imputation (MI), MI was consistently more accurate than SI within the same method (e.g. RSOV-MI performed better than RSOV-SI).

In general, the sampling methods (notably RSOV and hotdeck) performed less well for this particular case study. Across all mechanisms and missingness levels, the best-performing methods were most often RF and MM-MI (Tables 3 and 4). Other successful methods were PCA/MCA, especially for the ordered age data, and PMM-MI.

**Table 3:** The best performing methods for each imputation method, missingness mechanism, and missingness level (see Supplementary Information Table B for complete results); rel_MAE = relative mean absolute error, NRMSE_col_mean_sq = normalized root mean square error column mean square, NRMSE_tot_sd = normalized root mean square error total standard deviation, MCAR = missing completely at random, MAR = missing at random, MNAR = missing not at random, RF = Random Forest, RSOV = random samples from observed values, PMM = predictive mean matching, MM = multi-method approach, SI = single imputation, MI = multiple imputation

Missingness Mechanism	Data Type	5% Missingness	10% Missingness	20% Missingness	30% Missingness	40% Missingness
MCAR	Binary	MM-MI	MM-MI	MM-MI	MM-MI	MM-MI
	Unordered	RF	MM-MI	RF, MM-MI	RF	PMM-MI
	Ordered—Age	PCA/MCA	PCA/MCA	PCA/MCA	PCA/MCA	PCA/MCA
	Ordered—Severity	RF	RF	RF	RF	RF, PMM-MI
	Numeric—rel_MAE	RF	MM-MI	MM-MI	MM-MI	MM-MI
	Numeric—NRMSE_col_mean_sq	RF	MM-MI	MM-MI	MM-MI	MM-MI
	Numeric—NRMSE_col_mean_sq	RF	MM-MI	MM-MI	MM-MI	MM-MI
MAR	Binary	MM-MI	MM-MI	RF, MM-MI	MM-MI	PMM, MI
	Unordered	MM-MI	MM-MI	RF	PMM, MI	RF
	Ordered—Age	MM-MI	PCA/MCA	PCA/MCA	RSOV-MI	PCA/MCA
	Ordered—Severity	RF	RF	RF	RF	RF
	Numeric—rel_MAE	MM-MI	MM-MI	MM-MI	MM-MI	MM-MI
	Numeric—NRMSE_col_mean_sq	MM-MI	MM-MI	MM-MI	RF	MM-MI
	Numeric—NRMSE_tot_sd	RF	MM-MI	MM-MI	MM-MI	MM-MI
MNAR	Binary	PMM-MI	PMM-MI, MM-MI	PMM-MI, MM-MI	PMM-MI, MM-MI	PMM-MI, MM-MI
	Unordered	RF	PMM-SI	PMM-MI, MM-MI	PMM-MI, MM-MI	PMM-MI, MM-MI
	Ordered—Age	PCA/MCA	PCA/MCA	RF	PCA/MCA	RF
	Ordered—Severity	RF	MM-MI	PMM-MI	PMM-MI	RF
	Numeric—rel_MAE	RF	RF	MM-MI	MM-MI	MM-MI
	Numeric—NRMSE_col_mean_sq	RF	RF	MM-MI	MM-MI	MM-MI
	Numeric—NRMSE_tot_sd	RF	RF	RF	PMM-MI	MM-MI

**Table 4:** The out-of-bag (OOB) error results for the random forest imputations. Data includes a normalized root mean square error (NRMSE) for numeric data and the proportion of correctly imputed values (PFC) for categorical data; MCAR = missing completely at random, MAR = missing at random, MNAR = missing not at random

Missingness Mechanism	OOB Error	5%	10%	20%	30%	40%
MCAR	NRMSE	0.049	0.050	0.054	0.058	0.039
	PFC	0.151	0.159	0.167	0.170	0.167
MAR	NRMSE	0.049	0.051	0.053	0.040	0.040
	PFC	0.163	0.160	0.159	0.155	0.173
MNAR	NRMSE	0.050	0.049	0.053	0.054	0.059
	PFC	0.158	0.153	0.156	0.141	0.162

## Discussion

Looking first at the missingness mechanisms, Table 2 highlights the most effective mechanism for each imputation method; however, many findings were very similar between mechanisms. Furthermore, the averages are broad calculations that do not account for potentially important variations between the missingness levels. For more detailed values with regard to each calculation, see Supplementary Information Table C.

Overall, one notable pattern is that MCAR and MAR tended to impute better than MNAR for categorical data; however, imputations of continuous data were often more accurate for MNAR data. One possible explanation for this finding could be the structure of categorical data itself, which is by definition discrete with limited possibilities that could then allow for patterns to be more easily identified when the missingness is random. On the other hand, the missingness inherent in MNAR may actually be informative in some cases, revealing patterns that the more flexible continuous data can pick up on more easily. Indeed, this would be an interesting pattern to explore further in future studies.

Two outlying findings are also worth a second look. First is the exceptional result for all imputation methods imputing the ordered data for Age Group at 30% missingness for MAR data. It is possible that the data structure is such that 30% MAR missingness is the sweet spot for balancing information linked to random missingness based on observed samples and the imputation methods’ abilities to capture patterns in the data. It could also be that 30% missingness aligns with the imputation assumptions. This finding could also be due to a flaw in the code or the data; however these datasets were verified and re-calculated with the same results. Because no other variables illustrated this same anomaly, the most likely explanation probably involves the random, dependent variable upon which the MAR 30% dataset was generated. This random variable was probably directly correlated to age, thereby making this variable’s imputation exceptionally accurate. Second are the results of the LASSO select linear regression for continuous data (part of the MM-MI method) for lower levels of missingness (Figs. 3, 4, 5, 6, 7 and 8). These calculations found that imputation accuracy actually increased alongside missingness for MNAR continuous data, which is a surprising observation. One possible explanation could be the combination of LASSO’s method of shrinkage and feature selection with the “nonignorable” pattern of MNAR missingness. As missingness increases, LASSO may be able to shrink the coefficients more accurately, especially when that missingness is non-random, thus providing even more information in its missingness.

The percentage of missingness is the primary consideration when dealing with imputation accuracy across all methods. However, the imputation quality also depends on the structure of the dataset – imputations will tend to be more accurate when the variables are related. With regard to datasets in biological anthropology, this could serve as an advantage because variables such as measurements tend to be correlated (e.g. tibia lengths are often proportional to femur and fibula lengths). This is perhaps especially important for the dimension reduction methods because they take into account the relationships between variables as well as between individuals. Therefore as missingness increases, these calculated relationships become less reliable (Audigier et al. 2016). The results of this study therefore support previous recommendations that imputations should not be performed on datasets with more than 30% missingness (see Serneels and Verdonck 2008), and ideally datasets should have less than 10%-15% missingness (also see Ginkel et al. 2014).

Each imputation method has various advantages and disadvantages (Table 5); therefore, the results presented in this paper are unique to the control dataset and may not hold for others with different parameters. Looking first at the sampling-based methods, these tended to be the least accurate, with the exception of PMM-MI. Such imputation methods, in particular hotdeck, can be an accurate and timely way to obtain imputed datasets; however, due to the nature of many sampling processes, they may tend to be more effective on Big Data datasets, which most current bioarchaeological datasets are not. Big Data is usually defined as having the three Vs (i.e. volume, velocity, and variety) to such an extent that standard databases and computers cannot handle it (De Mauro et al. 2016; Favaretto et al. 2020). Hotdeck in particular is not dependent on model-fitting, therefore it is sensitive to issues like overfitting and possible imprecise parameters common to parametric models, such as regression (Andridge and Little 2010). One advantage to all sampling-based methods is that imputed values are realistic as they have been observed elsewhere in the dataset. However, they often make assumptions about individual similarity, which might not be advisable for certain datasets.

**Table 5:** The advantages and disadvantages of the select imputation methods applied in this study

**Imputation Method**	**R package**	**Advantages**	**Disadvantages**	**Sources**
Random Forest (RF)	missForest	Works well with high-dimensional data Considers relationships between variables Works well with complex, non-linear datasets Robust to noise Non-parametric Does not assume variable linearity Provides OOB and/or PFC error	Lack of interpretability Algorithm (not a model) Better for large datasets Assumes MCAR or MAR	Hong and Lynn 2020 Stekhoven and Bühlmann 2012
PCA/MCA	missMDA	Maximizes data preservation Maintains data structure Considers similarities between individuals and variables	Not hollistic—Does not consider relationship between categorical and continuous data Less ideal if variables include multiple data types Less reliable when relationships between variables are very non-linear (PCA) or very unassociated (MCA) Less performative for datasets with complex relationships between variables	Audigier et al. 2016
Factorial Analysis for Mixed Data (FAMD)	missMDA	Maintains the data structure Hollistic Maximizes data preservation Considers similarities between individuals and variables Does not require linear relationship between variables Accurate for rare categories	Prefers variable linearity Better with large datasets Less ideal if variables include multiple data types Less reliable when relationships between variables are very non-linear	Audigier et al. 2016
Hotdeck	VIM	Preserves distribution (imputes realistic values) Less sensitive to overfitting Fast and simple	Not ideal for small datasets (Big Data oriented) Does not consider complex variable relationships Sensitive to donors	Andridge and Little 2010 Joenssen and Bankhofer 2012 Kowarik and Templ 2016
Random Samples from Observed Values (RSOV)	mice	Computationally fast Simple to understand and apply Preserves distribution (imputes realistic values) Doesn’t require covariates	Does not consider data patterns or take into account relationships Less ideal for MAR data, not ideal for MNAR data Not ideal for categorical data, especially when many variables exist	van Buuren & Groothius-Ousdhoorn 2011
Predictive Mean Matching (PMM)	mice	Preserves distribution (imputes realistic values) Robust to model misspecifications Suitable for variables with non-normal distributions Considers patterns in the data	Requires a similar donor, not ideal for small datasets Relies on mean, therefore sensitive to outliers Computationally demanding (can be time-consuming) Not ideal for higher levels of missingness Not ideal for non-linear relationships or data with high-dimensionality	Bailey et al. 2020 Kleinke 2018 Morris et al. 2014 van Buuren 2018
**Multi-Method (MM) Approach—From the R package "mice"**				
**Imputation Method**	**Data Type**	**Advantages**	**Disadvantages**	**Sources**
LASSO Select + Logistic Regression	Binary	Good for high-dimensionality Interpretability (shrinking coefficients simplifies the model) Deals with multicollinearity (can separate effects of predictors) Regularization (reduces risk of overfitting)	Assumes a linear relationship between variables Often requires proper parameter tuning Computationally demanding (likely due iteratively reweighted least squares (IRLS) procedure) Could be sensitive to problems of perfect prediction	Austin & van Buuren 2023 Tibshirani 1996 White et al. 2012
Polytomous Logistic Regression (PLR)	Unordered	Can handle multiple categories Captures relationships between categories	Documented robusticity issues (might not handle outliers well) Might not work well on smaller datasets, especially if multiple categories are involved Difficult to interpret	Miron et al. 2022 van Buuren 2018
Proportional OddsModel (POM)	Ordered	Specific to ordered data – can assess proportional odds Model flexibility (specifically, predictor variable relationships) Works well as multiple imputation	Might not work well on smaller datasets Complex and difficult to interpret Could be sensitive to assumptions (e.g. proportional odds) – Log-odds independent of outcome category*	Hosmer et al. 2013 Liu et al. 2018 van Buuren 2018
LASSO Select + Linear Regression	Continuous	Good for high-dimensionality Interpretability (shrinking coefficients simplifies the model) Deal with multicollinearity (can separate effects of predictors) Regularization (reduces risk of overfitting)	Assumes a linear relationship between variables Reducing the wrong coefficients could lead to a loss of important information Often requires proper parameter tuning Could be sensitive to the scale of predictor variables	Austin & van Buuren 2023 Tibshirani 1996 White et al. 2012

*The R package “mice”, function “polr” (used in this study), applies an ordered logit model, which is a type of proportional odds model that does not assume that the odds of moving from between categories is proportional

Looking next at the PCA/MCA and FAMD methods, one major advantage is their ability to take into account the relationship between both individuals and variables, and MCA imputation is particularly effective for multivariate data (Audigier et al. 2016; Nienkemper-Swanepoel et al. 2023). However, while considering variable relationships could be a valuable feature, this is often done by looking at pairs of variables. Therefore, datasets with high-dimensionality and complex relationships between multiple variables, as often seen in bioarchaeology, could render the imputations more difficult (Audigier et al. 2016). Indeed, this study found FAMD to perform generally quite poorly compared to other methods. In this case, simplifying the analyses by separating the continuous and categorical data as with PCA/MCA could be an advantage, as seen in this case study.

The two top-performing methods across all criteria were RF and MM-MI. One primary advantage to RF is its capacity to calculate the OOB (out-of-bag error). The OOB is a measure of prediction error, meaning that RF can estimate its own imputation accuracy (Breiman 2001; Stekhoven and Bühlmann 2012). Overall, RF works well on complex datasets with high-dimensionality, making it an attractive choice for many bioarchaeological analyses. In R, it is also simple to perform and does not require tuning numerous parameters. However, these aspects also make it difficult to interpret, meaning it could be difficult to understand how RF arrived at its decisions. With regard to MM-MI, applying various methods according to data type, while more complex theoretically and computationally, allows the researcher to adapt their imputations accordingly. This case study showed that even though the MM-MI did not necessarily take into account the relationships between all variables, applying a “best method” to each data type still managed to outperform other methods.

Lastly, natural limitations occur with SI methods, mainly they are limited in their ability to assess the accuracy of the imputed value based on the observed values; therefore statistical analyses on the complete dataset will underestimate estimator variability (Audigier et al. 2016). Indeed, this study found that the adapted MI method always outperformed SI for the same method, and since resources such as mice are so readily available and user-friendly, this seems to be an advisable step to help maximize the accuracy of data imputation. However, the purpose of the adapted MI method was to achieve a single, complete dataset; for studies looking to answer specific statistical questions, the original MI protocol within mice is preferable (van Buuren and Groothuis-Oudshoorn 2011).

This study is not suggesting the presence of bad or unreliable imputation methods, simply methods that worked better than others for this unique dataset. However, it is likely that many datasets in bioarchaeology contain similar variables with similar parameters and the results found here may be broadly applicable. When choosing an imputation method, it is essential to understand the advantages and disadvantages of each, the nature of the dataset in question, and the research goals. In this regard, it is the hope that this work will be able to serve as a general, starting guideline for future research.

### Limitations

The choice of an effective imputation method varies between datasets as it depends on factors such as missingness level and mechanism, data type, and research goal. When working with imputation models, it is necessary to consider the possible effects of overfitting – when the model works well on training data but does not generalize well to a new dataset. Additional controls such as splitting the datasets into designated training and test sets (hold-out validation) or separately training and testing the model on different sub-groups of the dataset (cross-validation) could serve as important controls for assessing general imputation performance.

By the time a researcher is deciding whether to use imputation and selecting the most appropriate method, it is important that they have already gone through the proper steps to produce a quality dataset, including ensuring the data collected are representative of the population being studied and testing for inter and intraobserver error and removing inconsistent variables. Imputing inconsistent or unrepresentative data can amplify existing problems in the dataset and produce incorrect results (Pang et al. 2022). An essential point from this study is that imputation methods are not “one-size-fits-all”, and indeed all datasets are different. Therefore, this study can present the methods that worked best for this particular control dataset, but this is not necessarily representative of all bioarchaeological datasets; for example, the control dataset had relatively few variables especially with regard to the number of samples. Additional testing using real bioarchaeological data is needed to ascertain how the results found here compare with those from varying archaeological contexts. Lastly, a single dataset may contain more than one type of missingness. This potential limitation would require additional efforts from the researcher, with possible solutions including modifying the research question, creating multiple datasets, and applying a multi-method approach adapted to varying types of missingness.

In order to simplify the procedures, and because this study was not looking to use the sample data to answer a particular research question, this study used many of the default settings included in each imputation method. However, when looking at a focused research question, additional parameters, notably which variables to include as predictors and the imputation order (i.e. it is possible that imputation accuracy changes depending on which samples or variables are imputed first) (van Buuren 2018), become vital considerations for imputation performance.

It is also necessary to consider computation time. One of the best performing methods for this case study, MM-MI, was also the most time consuming (imputations using this method took several hours on a personal laptop with 16 GB of RAM). Theoretically, increasing iterations and the number of multiple imputations would also increase imputation accuracy, but it also increases computational resources. With such computation times, MM-MI in particular (also PMM-MI and RSOV-MI) may not be feasible for even larger datasets or certain objectives.

Lastly, this study did not include a comprehensive list of all imputation methods for mixed data, nor did it include a comprehensive list of all packages and functions in R capable of performing imputations. Indeed, this work is intended as a starting point for future bioarchaeology studies looking to apply data imputations to mixed datasets, as well as contribute to discussions on the necessity of understanding bioarchaeological datasets and their analytical and interpretative capacity.

## Conclusion

Imputation methods serve as a valuable tool for data analysis in bioarchaeology due to the prevalence of missing data. In particular, imputations of mixed datasets can be an excellent solution allowing for improved multivariate analyses and data modeling. The most important factor for accurate imputation across all methods is the amount of missingness; the best way to improve imputation performance is to reduce missingness as much as possible. Each imputation method has advantages and disadvantages as well as varying functions. It is therefore important to understand each individual dataset, specifically the type of data and missingness mechanism, as well as the particular research goals. In general, this study found random forest imputation to be a robust method, and the calculation of an OOB error is an excellent resource for assessing imputation success. The multi-method, multiple imputation approach also performed very well, underlining the potential power of imputing data according to data type rather than through a holistic sampling method for mixed data. Lastly, the sampling-based methods, in particular RSOV and hotdeck, tended to perform the poorest. However, this does not mean that there are “bad” imputation methods, simply methods that are better adapted to a given dataset and research question. Each dataset has a unique structure that needs to be thoroughly understood in order to choose the best imputation method to maximize performance.

## Supplementary Information

Below is the link to the electronic supplementary material.

Supplementary file1 (XLSX 126 KB)

Supplementary file2 (XLSX 55 KB)

Supplementary file3 (XLSX 51 KB)

## Data Availability

No datasets were generated or analysed during the current study.

## Footnotes

1 The notation varies between “MNAR” and “NMAR” based on the publication. In the R package “missMethods”, it is displayed as “MNAR”, which is the notation used in this paper.
